# Postoperative Outcomes and Complication Profile Following Thyroidectomy: A Retrospective Analysis

**DOI:** 10.7759/cureus.97354

**Published:** 2025-11-20

**Authors:** Ramsha Shahid Khan, Hassan Iqbal, Taimoor Tahir, Chaudhary Adeel Ahmad, Paras Fatima, Ali Ahmed Sohail, Danyal Zahoor

**Affiliations:** 1 General Surgery, District Headquarters Teaching Hospital, Narowal, PAK; 2 General Surgery, District Headquarters Hospital, Sheikhupura, PAK; 3 General Surgery, Pakistan Institute of Medical Sciences, Islamabad, PAK; 4 General Surgery, Mayo Hospital, Lahore, PAK; 5 General Surgery, Shalamar Hospital, Lahore, PAK; 6 Anesthesiology, Shalamar Hospital, Lahore, PAK; 7 General Surgery, Jam Ghulam Qadir Civil Hospital, Hub, PAK

**Keywords:** hypocalcemia, multinodular goiter, recurrent laryngeal nerve injury, surgical outcomes, thyroidectomy

## Abstract

Background

Although thyroidectomy is a common surgical procedure for benign and malignant thyroid diseases, it remains associated with risks such as hypocalcemia and recurrent laryngeal nerve injury. Understanding the frequency and predictors of these complications is essential to improving surgical safety and patient outcomes. This study aimed to evaluate the surgical outcomes and frequency of complications following thyroidectomy in a tertiary care setting.

Methodology

This retrospective, observational, descriptive study was conducted at Shalamar Hospital, Lahore, Pakistan, from May 2022 to May 2025. A total of 215 patients who underwent thyroidectomy were included in the study. Data were extracted retrospectively from hospital records, operative notes, and discharge summaries. A structured data collection form was used to record patient demographics (age, gender), indication for surgery (benign or malignant pathology), type of thyroidectomy performed, intraoperative findings, and postoperative outcomes.

Results

Of the 215 patients included, 176 (81.9%) were female and 39 (18.1%) were male, with a mean age of 42.8 ± 11.3 years. Total thyroidectomy was performed in 129 (60.0%) patients, hemithyroidectomy in 54 (25.1%), and subtotal thyroidectomy in 32 (14.9%). Histopathology revealed benign lesions in 134 (62.3%) and malignant lesions in 81 (37.7%) cases. Postoperative complications were observed in 59 (27.4%) patients, with transient hypocalcemia being the most frequent in 39 (18.1%), followed by recurrent laryngeal nerve injury in 14 (6.5%) and wound-related issues. Complication rates were significantly higher in patients undergoing total thyroidectomy (p < 0.05) and those with malignant pathology (p = 0.031). The mean hospital stay was 3.1 ± 1.4 days, which was significantly longer in patients with complications (p < 0.001).

Conclusions

While thyroidectomy remains an effective treatment for benign and malignant thyroid diseases, the observed complication rate indicates that surgical safety can be further optimized. The relatively high overall complication rate highlights the importance of comprehensive preoperative assessment, precise surgical technique, and structured postoperative monitoring. Strengthening surgeon training, adopting intraoperative nerve monitoring, and standardizing institutional protocols may help reduce complications and improve patient outcomes in similar healthcare settings.

## Introduction

Thyroidectomy is a commonly performed surgical procedure worldwide for the treatment of a variety of thyroid disorders, including multinodular goiter, Graves’ disease, and thyroid malignancies [1]. The operation may involve lobectomy, subtotal, or total thyroidectomy, with the extent determined by disease pathology, risk of malignancy, and the surgeon’s judgment. Thyroidectomy is regarded as safe; however, there are several significant postoperative complications related to this procedure, which may influence short-term recuperation as well as the health-related quality of life in the longer term [2]. The complications that are most commonly reported after thyroid surgery include transient or permanent hypocalcemia, injury to the recurrent laryngeal nerve (RLN), hemorrhage, wound infection, seroma formation, and compromised airway [3]. Particularly, hypocalcemia occurs as a result of accidental injury, devascularization, or excision of the parathyroid glands and is more frequent after a complete thyroidectomy. Whereas a hypocalcemia episode can be treated with calcium supplementation, permanent hypoparathyroidism might become a lifelong exposure to calcium analogs and vitamin D analog intake, which adversely impacts the health of the patient [4]. Another significant concern is that RLN injury may cause hoarseness, voice fatigue, aspiration, or, if bilateral, airway obstruction. The reports of transient RLN palsy incidences report anything between 1% and 5%, with permanent injury being less than 1% in high-volume centers [5].

Hemorrhage, though uncommon (0.3-1.6%), can be life-threatening due to airway obstruction, necessitating urgent recognition and re-exploration [6]. Postoperative morbidity is also associated with other complications, including surgical site infection, seroma, chyle leak (in major neck dissections), and thyrotoxic crisis (especially in uncontrolled thyrotoxicosis). Such complications are different and dependent on patient-related factors, the nature of the disease, and the experience of the surgeon. Globally, perioperative and intraoperative nerve monitoring (IONM) has minimized the danger of complications considerably [7]. Nonetheless, in low and middle-income nations (LMICs), shortages of resources, delays in diagnosis, and inconsistencies in surgical skill may contribute to a higher rate of surgical complications [8]. Moreover, poor access to intraoperative adjuncts such as harmonic scalpels, hemostatic agents, and real-time nerve monitoring is another contributor to increased risk, and local data would be of great assistance in improving surgical practice [9]. Complication rates and outcomes of thyroidectomy have been reported in several studies in developed countries; however, there is a relative lack of thorough information from LMICs on thyroidectomies, most specifically in South Asia [10]. In Pakistan, the burden of thyroid diseases that must be managed surgically is still high: a condition of endemic goiter and thyroid nodules is observed. Nonetheless, there is a low level of reporting postoperative outcomes in local surgical centers, and institutional audits are not published very often. Morbidity and mortality rates are dropping after operations due to improved treatment and surgeon expertise [11]. Nevertheless, severe hypoparathyroidism, hypocalcemia, postoperative bleeding, and damage to the RLN may be some of the major side effects of thyroid surgery [12]. Risk factors of thyroidectomies have been researched according to some studies, including age, sex, and type of thyroid disease [13,14].

Objective

This study aimed to evaluate the surgical outcomes and frequency of complications following thyroidectomy in a tertiary care setting.

## Materials and methods

Study design and setting

This retrospective, observational, descriptive study was conducted in the Department of General Surgery at Shalamar Hospital, Lahore, Pakistan, over a three-year period from May 2022 to May 2025.

Study population and sampling

A total of 215 consecutive patients who underwent thyroidectomy during the study period were included using a non-probability consecutive sampling method. The sample size was determined based on the average annual volume of thyroid surgeries performed at the institution, ensuring adequate power to detect a 10% difference in postoperative complication rates, assuming a baseline rate of 25%, a 95% confidence level, and a 5% margin of error.

Inclusion and exclusion criteria

Patients aged ≥18 years who underwent total, subtotal, or hemithyroidectomy for benign or malignant thyroid disease with complete medical and surgical records were included. Exclusion criteria were (1) previous thyroid surgery (re-operative cases), (2) pre-existing RLN palsy or parathyroid disorders, and (3) incomplete documentation or inadequate follow-up (<6 months).

Preoperative evaluation

All patients underwent detailed preoperative assessment, including thyroid function tests, neck ultrasonography, and fine-needle aspiration cytology when indicated. Preoperative factors such as thyroid size/weight (from operative notes or pathology reports), substernal extension, comorbidities (documented using the American Society of Anesthesiologists (ASA) physical status classification), and prior neck surgery were recorded. Serum vitamin D and calcium levels were measured in most cases to guide perioperative supplementation.

Surgical technique

All operations were performed under general anesthesia using a standard Kocher’s collar incision. For parathyroid identification, systematic identification and preservation of parathyroid glands were done whenever possible; glands with compromised vascularity were autotransplanted into the sternocleidomastoid. RLN identification was done using routine visualization and preservation of the RLN along the tracheoesophageal groove; IONM was not available. Hemostasis was achieved using bipolar cautery and vessel-sealing devices (LigaSure or Harmonic Scalpel) when available. Drain placement was performed selectively at the surgeon’s discretion, typically for large glands, extensive dissection, or intraoperative oozing. All procedures were performed or supervised by consultant general surgeons with over 10 years of endocrine surgery experience, assisted by senior residents.

Intraoperative variables

Operative time (skin incision to closure), estimated blood loss, number of parathyroid glands identified/preserved, drain placement, and the type of thyroidectomy performed (total, subtotal, or hemithyroidectomy) were recorded.

Postoperative management

Serum calcium was measured 12 hours post-surgery and daily until discharge. Selective calcium and vitamin D supplementation were administered based on biochemical or clinical evidence of hypocalcemia (serum calcium <8.0 mg/dL or symptoms such as perioral numbness, paresthesia, or positive Chvostek/Trousseau sign). Routine laryngoscopy was not performed in all patients, but was reserved for those with voice changes, dysphonia, or aspiration symptoms, acknowledging potential ascertainment bias. Criteria for discharge included stable vital signs, tolerance of oral diet, absence of bleeding, and serum calcium ≥8.0 mg/dL (or asymptomatic status with oral supplementation).

Definitions of complications

Transient hypocalcemia was defined as serum calcium <8.0 mg/dL resolving within six months with or without supplementation. Permanent hypoparathyroidism was defined as hypocalcemia persisting for more than six months or requiring lifelong supplementation. Transient RLN palsy was defined as a voice change with laryngoscopic confirmation resolving within six months. Permanent RLN palsy was defined as persistence beyond six months. Other complications (hemorrhage, seroma, infection, chyle leak) were defined according to standard surgical criteria. All postoperative complications were graded using the Clavien-Dindo classification for uniformity.

Follow-up protocol

Patients were followed up at two weeks, one month, three months, and six months postoperatively in outpatient clinics. Only patients with a minimum six-month follow-up were included in the final analysis. The median follow-up duration was 7.2 months (interquartile range = 6-9 months), with a loss-to-follow-up rate of 8.3%.

Data collection

Data were extracted retrospectively from hospital medical records, operative notes, histopathology reports, and postoperative follow-up documentation. A structured proforma ensured consistency in data recording. Variables included demographic details (age, gender), indication for surgery, type of thyroidectomy, histopathology findings, and postoperative outcomes. Postoperative complications assessed included transient or permanent hypocalcemia, RLN injury, postoperative hemorrhage requiring re-exploration, wound infection, seroma formation, and rare events such as thyrotoxic crisis or chyle leak. The primary outcome was the frequency and type of postoperative complications following thyroidectomy. Secondary outcomes included the relationship of complications with the type of surgery and disease pathology, and their effect on hospital stay.

Statistical analysis

Data were analyzed using IBM SPSS Statistics version 26 (IBM Corp., Armonk, NY, USA). Categorical variables (gender, surgery type, pathology, complications) were summarized as frequencies and percentages, while continuous variables (age, hospital stay) were presented as mean ± standard deviation. Group comparisons were performed using the chi-square test for categorical data and independent-samples t-tests for continuous variables. To identify independent predictors of postoperative complications, a multivariate logistic regression model was applied. Variables with p-values <0.10 in univariate analysis (age, gender, comorbidities/ASA class, procedure type, pathology, surgeon experience) were entered into the model. Adjusted odds ratios (AORs) with 95% confidence intervals (CIs) were reported. A subgroup analysis was also performed: complication rates stratified by procedure type (total, subtotal, hemi), and complication rates among malignant cases stratified by histological subtype (papillary, follicular, medullary, others). A p-value <0.05 was considered statistically significant. Multiple comparisons were corrected using the Bonferroni method.

## Results

A total of 215 patients who underwent thyroidectomy during the study period were included in the analysis, with a mean age of 42.8 ± 11.3 years, predominantly female (176, 81.9%). The majority of cases involved benign thyroid disease at 134 (62.3%), while 81 (37.7%) had malignant pathology. Total thyroidectomy was the most commonly performed procedure in 129 (60%), followed by hemithyroidectomy in 54 (25.1%), and subtotal thyroidectomy in 32 (14.9%). Histopathological analysis confirmed benign lesions in 135 (62.8%) and malignant lesions in 80 (37.2%). Multinodular goiter was the leading indication for surgery in 98 (45.6%), followed by confirmed malignancy in 47 (21.9%), and suspicious thyroid nodules in 41 (19.1%) (Table 1).

**Table 1:** Demographic and clinical characteristics of patients (n = 215). Data are represented as mean ± SD or n (%).

Variable	Value
Age (years), mean ± SD	42.8 ± 11.3
Gender, n (%)	
Female	176 (81.9)
Male	39 (18.1)
Thyroid disease type, n (%)	
Benign	134 (62.3)
Malignant	81 (37.7)
Surgical type, n (%)	
Total thyroidectomy	129 (60.0)
Hemithyroidectomy	54 (25.1)
Subtotal thyroidectomy	32 (14.9)
Histopathology, n (%)	
Benign	135 (62.8)
Malignant	80 (37.2)
Indications for surgery, n (%)	
Multinodular goiter	98 (45.6)
Suspicious thyroid nodule (Bethesda III–V)	41 (19.1)
Graves’ disease	26 (12.1)
Confirmed thyroid malignancy	47 (21.9)
Cosmetic or pressure symptoms	3 (1.3)

Postoperative complications were observed in 59 (27.4%) patients. Transient hypocalcemia was the most frequent issue, affecting 39 (18.1%) cases, while permanent hypoparathyroidism developed in 8 (3.7%). RLN injury occurred in 6.5% of patients, with 10 (4.7%) being transient and 4 (1.9%) permanent. Other complications included postoperative hemorrhage in 5 (2.3%), wound infections in 6 (2.8%), and seroma formation in 7 (3.3%) (Table 2).

**Table 2:** Frequency of postoperative complications. Values are expressed as n (%).

Complication	n (%)
Any complication	59 (27.4)
Transient hypocalcemia	39 (18.1)
Permanent hypoparathyroidism	8 (3.7)
Transient recurrent laryngeal nerve injury	10 (4.7)
Permanent recurrent laryngeal nerve injury	4 (1.9)
Postoperative hemorrhage	5 (2.3)
Wound infection	6 (2.8)
Seroma formation	7 (3.3)

Total thyroidectomy was associated with the highest complication rate in 46 (35.6%), which was statistically significant (χ² = 7.82, p < 0.05), compared to hemithyroidectomy in 10 (18.5%) and subtotal thyroidectomy in 7 (21.9%). Patients with malignant pathology also had a significantly higher complication rate (29, 34.4%) compared to those with benign disease (30, 22.2%) (χ² = 4.65, p = 0.031) (Table 3).

**Table 3:** Complication rates based on type of surgery and pathology.

Group	Complication rate, N (%)	Test statistic	P-value
Type of surgery			
Total thyroidectomy	46 (35.6)	χ² = 7.82, df = 2	<0.05
Hemithyroidectomy	10 (18.5)		
Subtotal thyroidectomy	7 (21.9)		
Pathology type			
Benign pathology	30 (22.2)	χ² = 4.65, df = 1	0.031
Malignant pathology	29 (34.4)		

The overall mean hospital stay was 3.1 ± 1.4 days. Patients who developed complications had a significantly longer stay (4.2 ± 1.6 days) compared to those without complications (2.6 ± 1.1 days), with the difference being statistically significant (t = 5.61, p < 0.001) (Table 4).

**Table 4:** Mean length of hospital stay.

Group	Length of stay (days)	Test statistic	P-value
Overall mean stay	3.1 ± 1.4	–	–
With complications	4.2 ± 1.6	t = 5.61, df = 213	<0.001
Without complications	2.6 ± 1.1	–	–

Among the 80 malignant cases, papillary thyroid carcinoma was the most common histological subtype in 61 (76.3%), followed by follicular carcinoma in 9 (11.3%) and medullary carcinoma in 6 (7.5%). Poorly differentiated or anaplastic carcinoma was identified in 3 (3.7%) patients, while rare subtypes, including metastatic lesions, were found in 1 (1.2%) (Table 5).

**Table 5:** Histological subtypes among malignant thyroid cases (n = 80). Values are expressed as n (%).

Histological type	n (%)
Papillary thyroid carcinoma	61 (76.3)
Follicular carcinoma	9 (11.3)
Medullary carcinoma	6 (7.5)
Poorly differentiated/anaplastic	3 (3.7)
Others (e.g., metastasis)	1 (1.2)

On multivariate logistic regression, total thyroidectomy (AOR = 2.45, 95% CI =1.21-4.96, p = 0.013) and malignant pathology (AOR = 1.98, 95% CI = 1.01-3.89, p = 0.046) were independent predictors of postoperative complications. Age, gender, and ASA class were not statistically significant after adjustment (all p > 0.05). Surgeon experience (>10 years vs. <10 years) showed a protective but non-significant trend (AOR = 0.71, 95% CI = 0.36-1.42, p = 0.34) (Table 6).

**Table 6:** Multivariate logistic regression for predictors of postoperative complications. AOR = adjusted odds ratio; ASA =American Society of Anesthesiologists; CI = confidence interval

Variable	AOR	95% CI	P-value
Total thyroidectomy (vs. hemi/subtotal)	2.45	1.21–4.96	0.013
Malignant pathology (vs. benign)	1.98	1.01–3.89	0.046
Age >50 years	1.27	0.66–2.45	0.48
ASA ≥III	1.36	0.62–2.96	0.43
Surgeon experience >10 years	0.71	0.36–1.42	0.34

Among the 80 malignant cases, papillary carcinoma had a complication rate of 32.8%, follicular 33.3%, medullary 50%, and anaplastic 66.7%. Although higher rates were seen in aggressive histologies, the differences were not statistically significant (p = 0.09, χ² test), likely due to small subgroup sizes (Table 7).

**Table 7:** Complication rates by histological subtype (malignant cases).

Histological subtype	n (%)	Complication rate, n (%)
Papillary carcinoma	61 (76.3)	20 (32.8)
Follicular carcinoma	9 (11.3)	3 (33.3)
Medullary carcinoma	6 (7.5)	3 (50.0)
Anaplastic/Others	4 (5.0)	2 (50.0–66.7)

When benchmarked against international data (reported complication range of 15-30%), the overall 27.4% rate in this study falls at the upper acceptable limit. Permanent hypoparathyroidism (3.7%) and permanent RLN injury (1.9%) exceeded high-volume center averages (1-2% and <1%, respectively). However, this difference may reflect inclusion of advanced malignancies (37.7% of cohort), total thyroidectomy predominance (60%), and limited access to IONM or parathyroid autofluorescence tools.

## Discussion

This retrospective study evaluated surgical outcomes and postoperative complications among 215 patients undergoing thyroidectomy at a tertiary care center in Pakistan. These results demonstrate the overall safety of thyroidectomy while highlighting challenges that persist for endocrine surgeons in low-resource settings. The majority of patients were female (81.9%), consistent with international and regional epidemiological trends showing a higher prevalence of thyroid disease in women [15]. Total thyroidectomy was the most frequently performed procedure (60%), reflecting modern surgical practice favoring complete gland removal in multinodular goiter, Graves’ disease, and nodules suspicious for malignancy where preoperative exclusion of malignancy is not definitive [15]. Hemithyroidectomy (25.1%) and subtotal thyroidectomy (14.9%) were less common, reflecting a tailored approach depending on disease extent and intraoperative findings.

The overall postoperative complication rate was 27.4%, with transient hypocalcemia being the most frequent (18.1%). Permanent hypoparathyroidism occurred in 3.7% of cases, higher than international benchmarks (1-2%) but comparable to some South Asian reports (3-5%) [16]. RLN injury occurred in 6.5% of patients, with 1.9% permanent, exceeding rates reported in high-volume centers (<1%) [17]. Higher complication rates in our cohort may reflect a combination of factors: a larger proportion of total thyroidectomies, cases with malignant pathology, absence of IONM, and resource constraints common in regional settings. Postoperative hemorrhage was uncommon (2.3%) but remains potentially life-threatening, underscoring the importance of early recognition and prompt surgical intervention [18].

Patients undergoing total thyroidectomy and those with malignant pathology had significantly higher complication rates (35.6% and 34.4%, respectively), highlighting the importance of preoperative risk stratification. These findings are consistent with prior studies from Pakistan and South Asia, where aggressive surgery, substernal extension, and hypervascular thyroid glands were associated with increased postoperative hypocalcemia and RLN injury [19]. The mean length of hospital stay was 3.1 ± 1.4 days, but it was significantly longer in patients with complications (4.2 ± 1.6 days vs. 2.6 ± 1.1 days, p < 0.001), reflecting both patient morbidity and increased resource utilization [20].

This study provides a critical appraisal of factors influencing complication rates. The retrospective design and single-center setting limit generalizability and introduce selection bias, as tertiary referral centers tend to manage more complex cases. Information bias is possible due to reliance on medical records, operative notes, and selective follow-up for laryngoscopy. Only patients with a minimum six-month follow-up were included, resulting in a modest loss-to-follow-up rate (8.3%), but ascertainment bias cannot be excluded. The lack of routine IONM and parathyroid hormone monitoring limited early detection and risk-adjusted analysis of complications.

Despite these limitations, this study contributes valuable regional data on thyroidectomy outcomes, which remain scarce in Pakistan. The findings have several clinical implications. First, risk-adjusted surgical planning is essential. Patients with malignant thyroid disease, large or substernal goiters, or anticipated extensive dissection should be counseled regarding higher complication risks. Second, enhanced intraoperative safety measures, including IONM, systematic parathyroid identification protocols, and selective use of vessel-sealing devices, may reduce RLN injury and hypoparathyroidism. Third, standardized postoperative monitoring with routine calcium measurement, structured discharge criteria, and selective laryngoscopy for symptomatic patients could improve early detection and management of complications. Fourth, training and capacity building are crucial; structured endocrine surgery training and supervision of junior surgeons are particularly important in low-resource settings to maintain safety benchmarks comparable to high-volume centers. Finally, future multicenter, prospective studies incorporating multivariate and risk-adjusted analyses are needed to establish benchmarks for the South Asian population, identify independent predictors of complications, and guide the development of evidence-based guidelines suitable for resource-limited healthcare settings.

## Conclusions

In our tertiary care institution, thyroidectomy demonstrated a complication rate of 27.4%, with transient hypocalcemia (18.1%) and RLN injury (6.5%) being the most frequent postoperative issues. Total thyroidectomy and malignant pathology were associated with higher complication rates, highlighting the influence of surgery type and underlying diagnosis on adverse outcomes. These findings underscore the importance of careful preoperative evaluation, risk stratification, meticulous surgical technique, systematic parathyroid preservation, and structured postoperative monitoring to minimize morbidity. Strengthening surgical training, considering intraoperative adjuncts such as nerve monitoring, and implementing standardized institutional protocols are essential to improving outcomes, particularly in resource-limited settings. Future multicenter, risk-adjusted studies with subgroup analyses by cancer stage, goiter size, and histological subtype, as well as evaluations of cost and quality of life, are warranted to establish benchmarks for the South Asian population and guide evidence-based strategies to further reduce postoperative complications.
